# Health Impact Bonds: Will Investors Pay for Intervention?

**DOI:** 10.1289/ehp.121-a45

**Published:** 2013-02-01

**Authors:** Rebecca Fairfax Clay

**Affiliations:** Rebecca Fairfax Clay has written for *EHP* since 1993. Her work has also appeared on National Public Radio and in the *Christian Science Monitor* and *The Environmental Forum*. In addition, she is the author of two children’s science books related to astronomy and space exploration.

With a 20% countywide pediatric asthma rate,^1^ Fresno, California, is the first U.S. community to test a health care funding strategy that could both reduce treatment costs and provide a financial incentive to investors.^2^ Based on growing national interest in social impact bonds (SIBs), experimental financing mechanisms to help tackle social issues, Fresno’s proposed health impact bond (HIB) would step in where governments often do not, by supporting efforts to reduce emergency department visits and hospital stays related to asthma.

As part of a pilot program, 200 local children with moderate to severe asthma will begin preventive care early in 2013. The children were chosen based on their asthma diagnosis as well as their related expenses based on medical claims data from MediCal (California’s Medicaid program)—the researchers are able to match each child’s diagnosis and treatment costs, giving them a clear idea of savings that could come at least in part from preventive care.

“Some of these children go to the emer-gency department almost every week,” says Rick Brush, founder and chief executive officer of Connecticut-based social enterprise Collective Health, which is overseeing the project. He says the focus in this pilot phase is on children who average 1.5 emergency department visits per year and end up staying in the hospital about half the time.

“The bond would pay for in-home interventions we know through numerous scientific studies improve patient health and lower the cost of care,” says Tony Iton, senior vice president for healthy communities at The California Endowment, a private health foundation. Iton says The California Endowment is spending $1.1 million to kick off Fresno’s HIB project.

Community health workers will visit the children’s homes, assess indoor triggers for asthma, and implement solutions that could include cleaning or replacing carpets, monitoring medication compliance, suggesting changes in behavior (for instance, not smoking around children), and removing dust, mold, and pests. The workers will follow up with monthly phone calls and quarterly home visits to provide further assistance and help ensure compliance with recommended practices. After a year the original claims data will be compared with the number of emergency department visits and hospitalizations as well as treatment costs after intervention.

**Figure:**
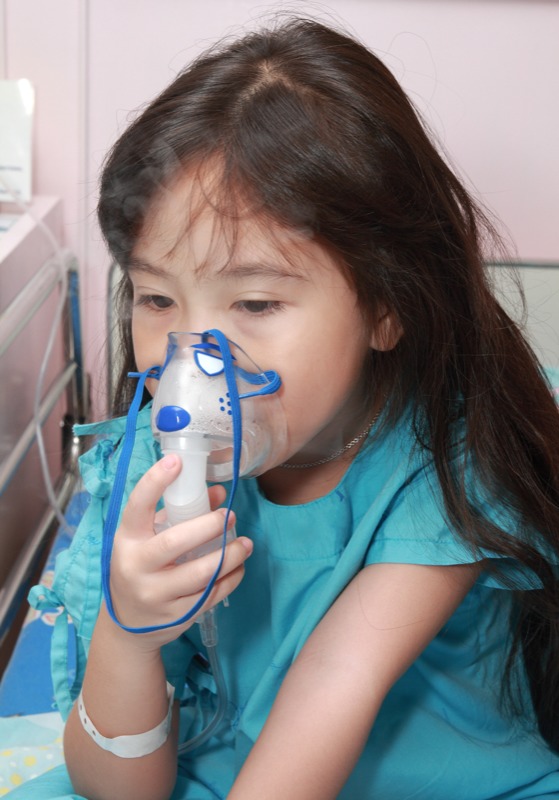
Emergency department visits can turn into hospital stays for asthma patients. © Phaitoon Sutunyawatchai/Shutterstock.com

According to the California Department of Public Health, approximately 157,000 people in Fresno have asthma.^3^ Using cost data from California,^3^ Florida,^4^ and Indiana,^5^ Brush estimated that asthma-related emergency department visits and hospitalizations set Fresno patients and insurers back about $35 million per year.^6^ (Adding indirect costs related to lost school and work days boosts his estimate to $87 million per year, he says.) So the potential for improved health care and savings could be significant.

“We project a thirty-percent reduction in emergency department visits and fifty-percent reduction in hospitalizations, with approximate net savings of five thousand dollars per child per year after paying for the intervention,” Brush says. “The HIB is raising capital for an intervention that would not necessarily have happened otherwise. We are investing in prevention to avoid emergencies and costs that are likely to happen if we do nothing.”

If the intervention is successful, insurers will pay out far less in health care reimbursements than they would have without the intervention. Investors will be repaid by the insurers a portion of the savings realized. Savings could also be used to provide preventive care to more patients.

After analyzing the actual savings from the pilot phase, Brush says his team will likely launch the bond in 2014, raising capital to scale up the effort to 1,000–3,500 children—the final number will depend in part on how many investment dollars are raised. Possible investors in the “pay-for-success” model could include foundations, government entities, private companies with health insurance programs (Whirlpool is planning a similar bond for its employees in Michigan^7^), individuals, and investment firms, he says.

With its greater emphasis on prevention, the U.S. Affordable Care Act^8^ should also support this kind of innovation, Brush says. By requiring health insurers to spend more premium dollars on care and fewer on administration and profit, the act might encourage insurers to invest in programs that reduce health care costs and provide a return.

SIBs are being tested to reduce teen criminal recidivism in New York City^9^ and homelessness in Massachusetts.^10^ Goldman-Sachs is putting nearly $10 million into the New York program—by helping keep teen offenders out of jail, the investment firm stands to profit from a drop in incarceration. And this is where some of the controversy lies. Critics say the two should not be linked—that charities and governments, rather than financial markets, should fund prevention.

Some SIB critics cite concerns about the pursuit of private profit over the common good, while others argue that a bond shown to work in one city may not work in another. Proponents, on the other hand, claim that private payers have more interest and latitude in saving money than charities and especially governments.^10^ Both Brush and Iton feel governments are hampered by politics and a fear of risk, and that the market will be willing to take that risk for potential profit—as long as they prove it can be done.

“Because asthma is eminently preventable,” adds Iton, “we want to find the most motivated partners we can. That is why we’re looking to the market to help improve health care and lower costs.”
